# Supplementary material for: Conformational Insights into the Control of CNF1 Toxin Activity by Peptidyl-Prolyl Isomerization: A Molecular Dynamics Perspective
Source: Int J Mol Sci. 2021 Sep 20;22(18):10129. doi: 10.3390/ijms221810129 (PMC8467853; doi:10.3390/ijms221810129)
Supplement: Supplementary file 1 [file ijms-22-10129-s001.zip › ijms-1355022-supplementary.pdf]

Supplementary information: Conformational insights into the control of CNF1 toxin activity by peptidyl-prolyl isomerization: a molecular dynamics perspective

Eléa Paillares[1,2], Maud Marechal[1], Léa Swistak[1,2], Landry Tsoumtsa Meda[1], Emmanuel Lemichez[1,*] and Thérèse E Malliavin[3,4,*]

September 20, 2021

[1] Unité des Toxines Bactériennes, UMR CNRS 2001, Institut Pasteur, 75015 Paris, France; elea.paillares@pasteur.fr (E.P.); maud.marechal@pasteur.fr (M.M.); lea.swistak@pasteur.fr (L.S.); landry.tsoumtsa-meda@pasteur.fr (L.T.M.)

[2] Université de Paris, Sorbonne Paris Cité, 75006 Paris, France

[3] Unité de Bioinformatique Structurale, UMR CNRS 3528, Institut Pasteur, 75015 Paris, France

[4] Institut Pasteur and CNRS USR 3756, rue du Dr Roux, Centre de Bioinformatique, Biostatistique et Biologie Intégrative, 75015 Paris, France

* Co-last authors, Emmanuel Lemichez and Thérèse Malliavin

† Correspondence authors : emmanuel.lemichez@pasteur.fr and therese.malliavin@pasteur.fr

Key words: *Escherichia coli*, CNF1; demaidase; X-Pro imide bond; proline isomerization; molecular dynamics; peptide docking

Methods

Self-organizing maps

A clustering approach, the **S**elf-**O**rganizing **M**aps (SOM), which is an artificial neural network (ANN) trained using unsupervised learning, was used to extract representative conformations from the molecular dynamics (MD) trajectories [1]. The conformations sampled along trajectories were encoded from the distances d_{ij} calculated between the n C_α atoms of the domain $CNF1^{CD}$, by diagonalizing the covariance matrix C:

$$C_{i,j} = \frac{1}{n}\sum_{k=1}^{n}\sum_{l=1}^{n}(d_{i,k} - \bar{d_i})(d_{l,j} - \bar{d_j}) \quad (1)$$

where $\bar{d_i} = \frac{1}{n}\sum_{j=1}^{n} d_{i,j}$. The information contained in the matrix C is equivalent to its four largest eigenvalues along with the corresponding eigenvectors. The eigenvalue and eigenvector descriptors are used to train a periodic Euclidean 2D self-organizing map (SOM), defined by a three-dimensional matrix.

The self-organizing maps were initialized with a random uniform distribution covering the range of values of the input vectors. At each step, an input vector is presented to the map, and the neuron closest to this input is updated. The maps are trained in two phases. During the first phase, the input vectors are presented to the SOM in random order to avoid mapping bias with a learning parameter of 0.5, and a radius parameter of 36. During the second phase, the learning and radius constants are decreased exponentially from starting values 0.5 and 36, respectively, during 10 cycles of presentation of all the data in random order. Once the

calculation of the SOM has been realized, the processed MD trajectory has been transformed into a map in which each pixel corresponds to a series of similar conformations of CNF1^{CD}. The conformations of the domain CNF1^{CD} corresponding to local maxima of homogeneity in the SOM map, are further used as representative conformations of the MD trajectory.

Results

Internal dynamics of CNF1

The coordinate root-mean-square deviation (RMSD, Å) calculated on the backbone heavy atoms with respect to the structure 1HQ0 displays a plateau at around 2 Å for all simulation conditions (Figure S1), which proves that the general architecture of protein structure does not drift much from the X-ray crystallographic structure. RMSD (Å) values have been also monitored on the loops L1 (residues 764-768), L2 (residues 789-795), L3 (residues 812-816), L4 (residues 833-838), L5 (residues 862-866), L6 (residues 884-889), L7 (residues 940-948), L8 (residues 964-970) and L9 (residues 996-1002) (Figure S2), the whole protein structure being superimposed on the whole X-ray crystallographic structure 1HQ0. Overall, the coordinate RMSD values do not display strong differences among the trajectories, except for L9. The largest RMSD values observed for L9 probably arise from its location close to the C terminal part of CNF1, and the second largest RMSD of L3 might be induced by the proximity of L3 to L9. Interestingly, the very mobile loop L6 has a symmetric position with respect to loop L8 (containing P968) with respect to the catalytic site. The loops L8 and L2 displays rather small RMSD values, but the lack of mobility of loops L4 and L5 is particularly striking, in agreement with the X-ray crystallographic structure in which these loops are less protruding than others. Otherwise, the loop L7 (residues 940-948) displays a very flat profile of median

RMSD distributions around 3 Å. This uniform mobility might be due to the presence of a short α helix (residues 945-948) located within L7, and interacting with the α helix formed by residues 921-932.

The *cis*/*trans* isomerization of P768 and P968 X-Pro imide bonds has an influence on internal mobility of loops (Figure S2). Indeed, the median values for L1 (containing P768) are larger in constrained trajectories in which only P968 is forced to be in *trans* conformation (orange boxes) than in other constrained trajectories (green and magenta boxes). Similarly, for loop L8 containing P968, the trajectories in which the P968 *trans* conformation is enforced (orange and green boxes) display median values somehow larger than the ones observed if P968 is kept in *cis* conformation (magenta boxes). In L9, neighbor of L8, the *trans* conformations imposed to P968 (blue and orange boxes) or to P768 (magenta boxes) induce median values among the largest ones.

References

[1] Bouvier, G., N. Desdouits, M. Ferber, A. Blondel, and M. Nilges, 2014. An automatic tool to analyze and cluster macromolecular conformations based on Self-Organizing Maps. *Bioinformatics* 31:1–3.

[2] Raveh, B., N. London, and O. Schueler-Furman, 2010. Sub-angstrom modeling of complexes between flexible peptides and globular proteins. *Proteins* 78:2029–2040.

Figure S1

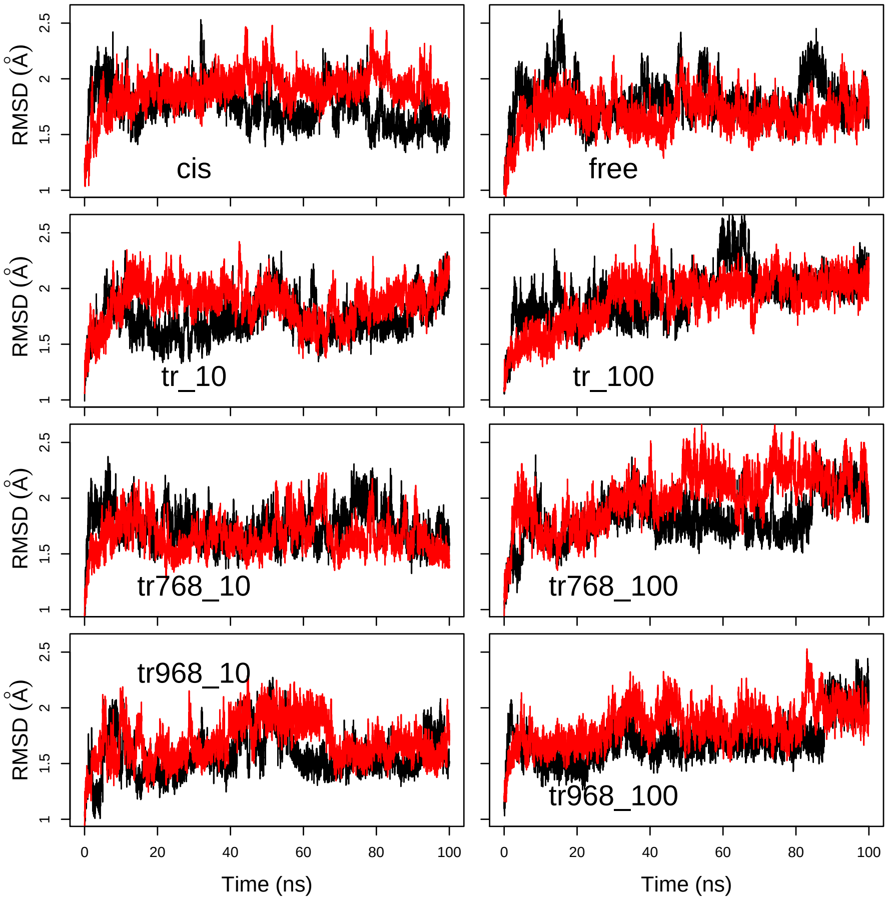

Figure S1. Coordinate RMSD (Å) of the backbone heavy atoms with respect to the X-ray crystallographic structure of CNF1^{CD} (1HQ0), calculated along all trajectories. For each trajectory, the black and red curves correspond to the two replicas R1 and R2.

Figure S2

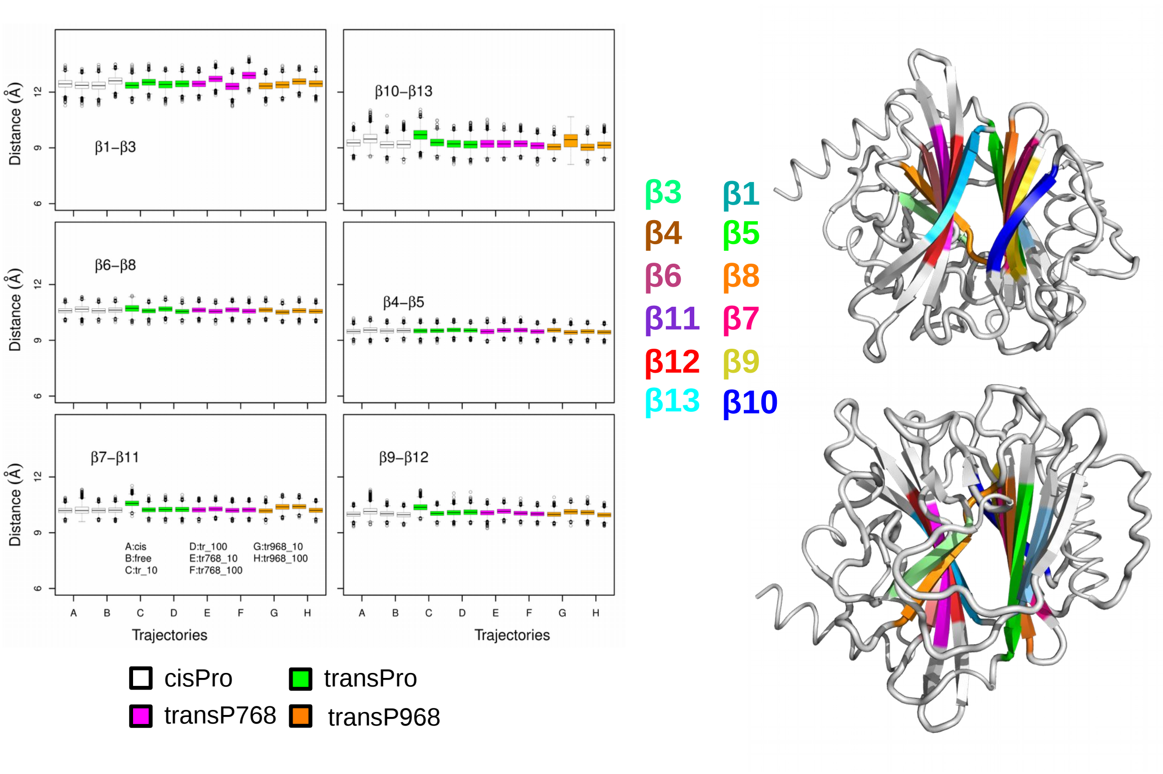

Figure S2. Average distances between the heavy backbone atoms of β strands: $\beta1$ (residues 759-762), $\beta3$ (residues 800-803), $\beta4$ (residues 829-835), $\beta5$ (residues 840-846), $\beta6$ (residues 856-862), $\beta7$ (residues 867-872), $\beta8$ (residues 876-882), $\beta9$ (residues 935-940), $\beta10$ (residues 957-962), $\beta11$ (residues 974-979), $\beta12$ (residues 989-994), $\beta13$ (residues 1008-1013). The distance distributions are plotted as box-plots colored in white (trajectories cis, free), green (trajectories tr_10, tr_100), magenta (trajectories tr768_10, tr768_100), orange (trajectories tr968_10, tr968_100). For each trajectory, the distributions of the two replicas are drawn. Two opposite views of the CNF1^{CD} structure with colored β strands are plotted in the right panel.

Figure S3

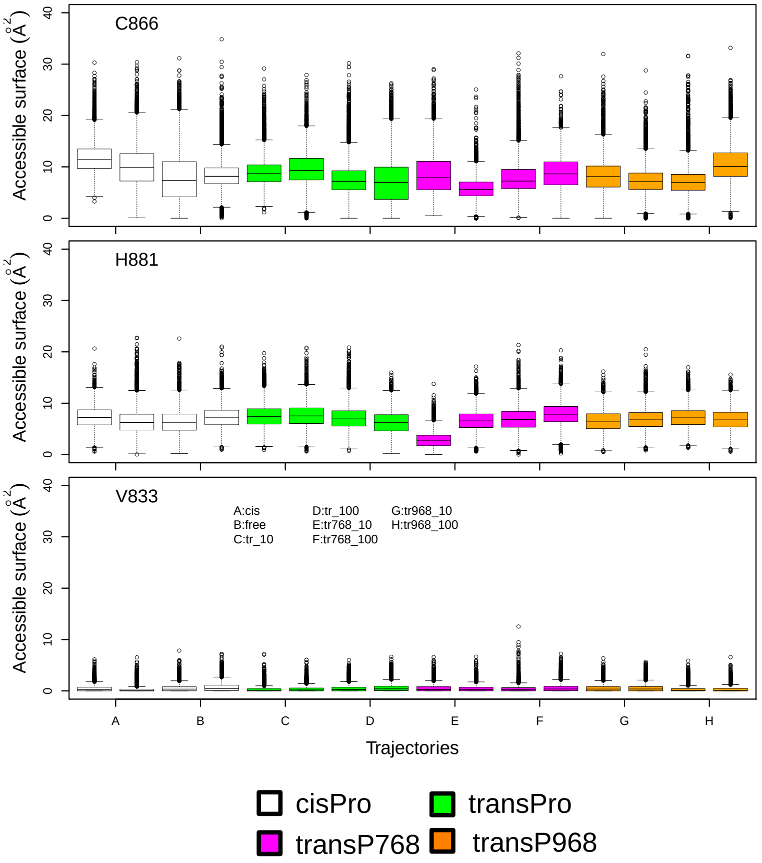

Figure S3: Variation of the accessible surface (Å^2) of C866 and H881 along the trajectories. For each trajectory, the distributions of the two replicas are drawn The surface distributions are plotted as box-plots colored in white (trajectories cis, free), green (trajectories tr_10, tr_100), magenta (trajectories tr768_10, tr768_100), orange (trajectories tr968_10, tr968_100).

Figure S4

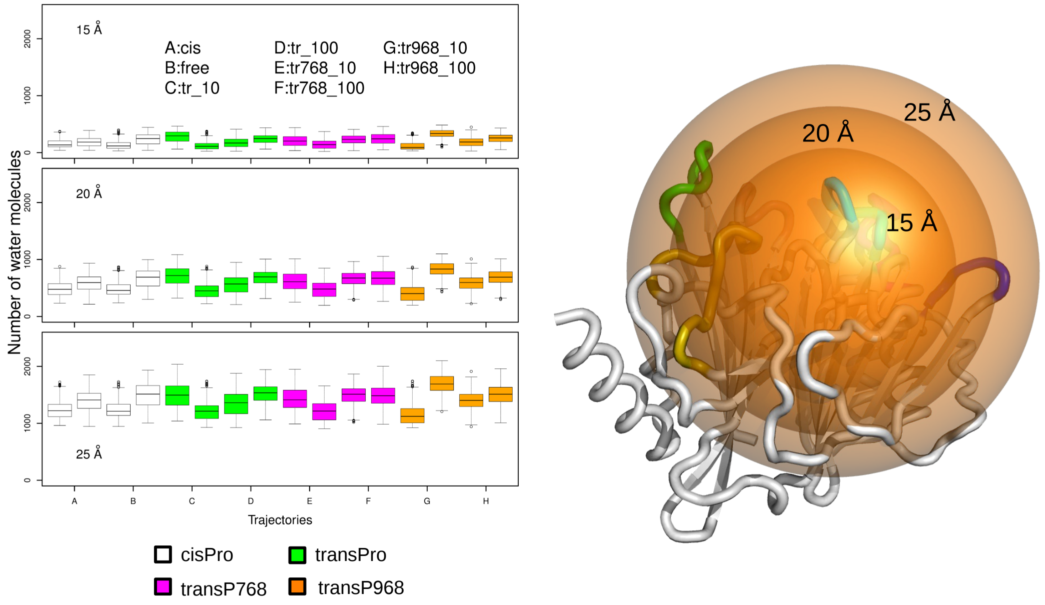

Figure S4: Distributions of the number of water molecules within several spheres centered to the geometric center of C866, with radii of 15 Å (top panels), 20 Å (intermediate panels) and 25 Å (bottom panels). The number of waters averaged on the trajectory time intervals of 90-100 ns (right column), are drawn on the two replicas. The number of water distributions are plotted as box-plots colored in white (trajectories cis, free), green (trajectories tr_10, tr_100), magenta (trajectories tr768_10, tr768_100), orange (trajectories tr968_10, tr968_100). Right panel: structure of CNF1^{CD} in cartoon with the spheres centered on Sγ atom of C866 and displaying the different radii used.

Figure S5

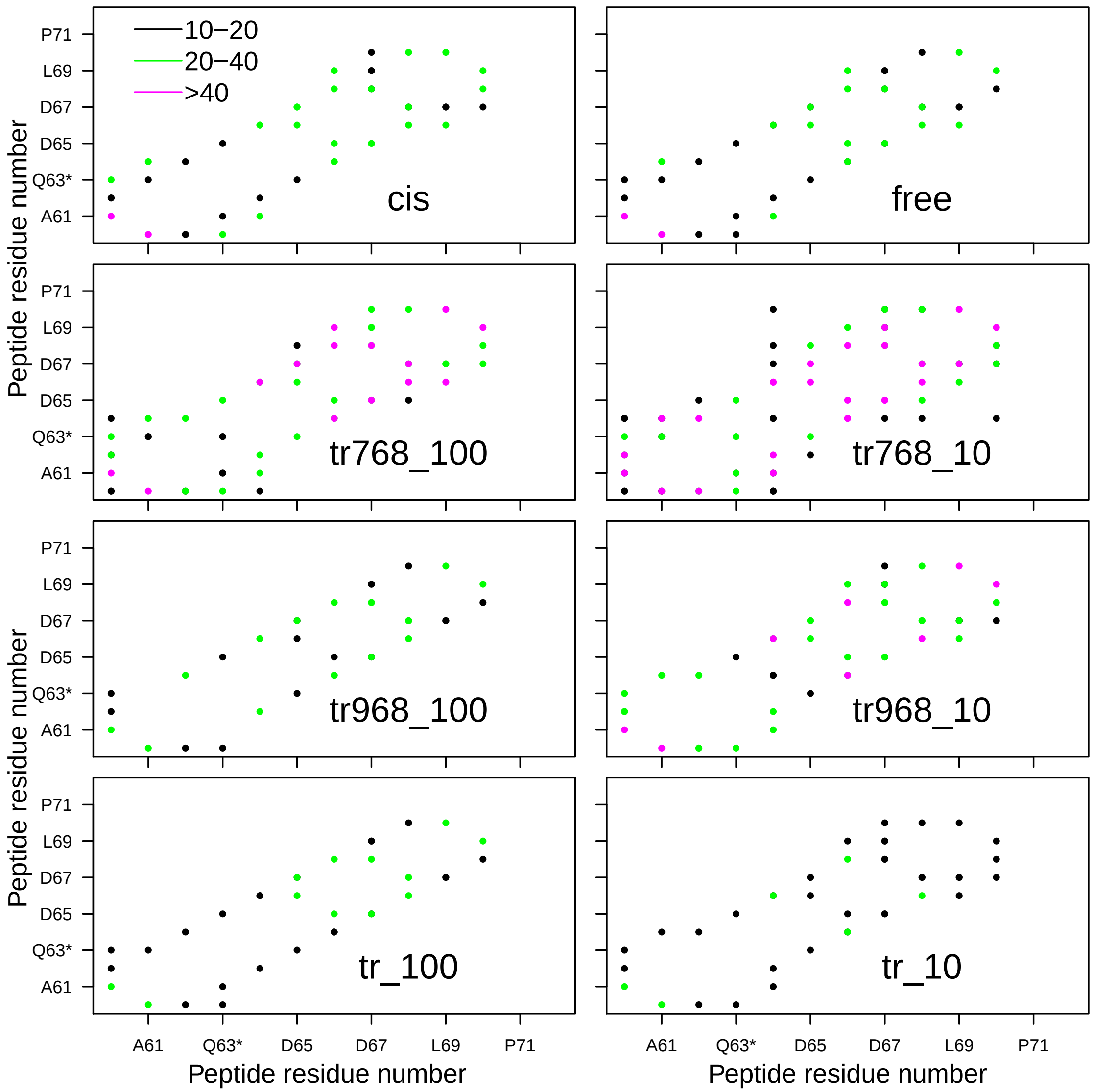

Figure S5: Contact maps of the hydrogen bonds observed within the SWII domain of RhoA in the various flexpepdock [2] runs. The points on the contact maps are colored depending on the number of hydrogen bonds observed: in the range 10-20 (black), in the range 20-40 (green), larger than 40 (magenta).

Figure S6

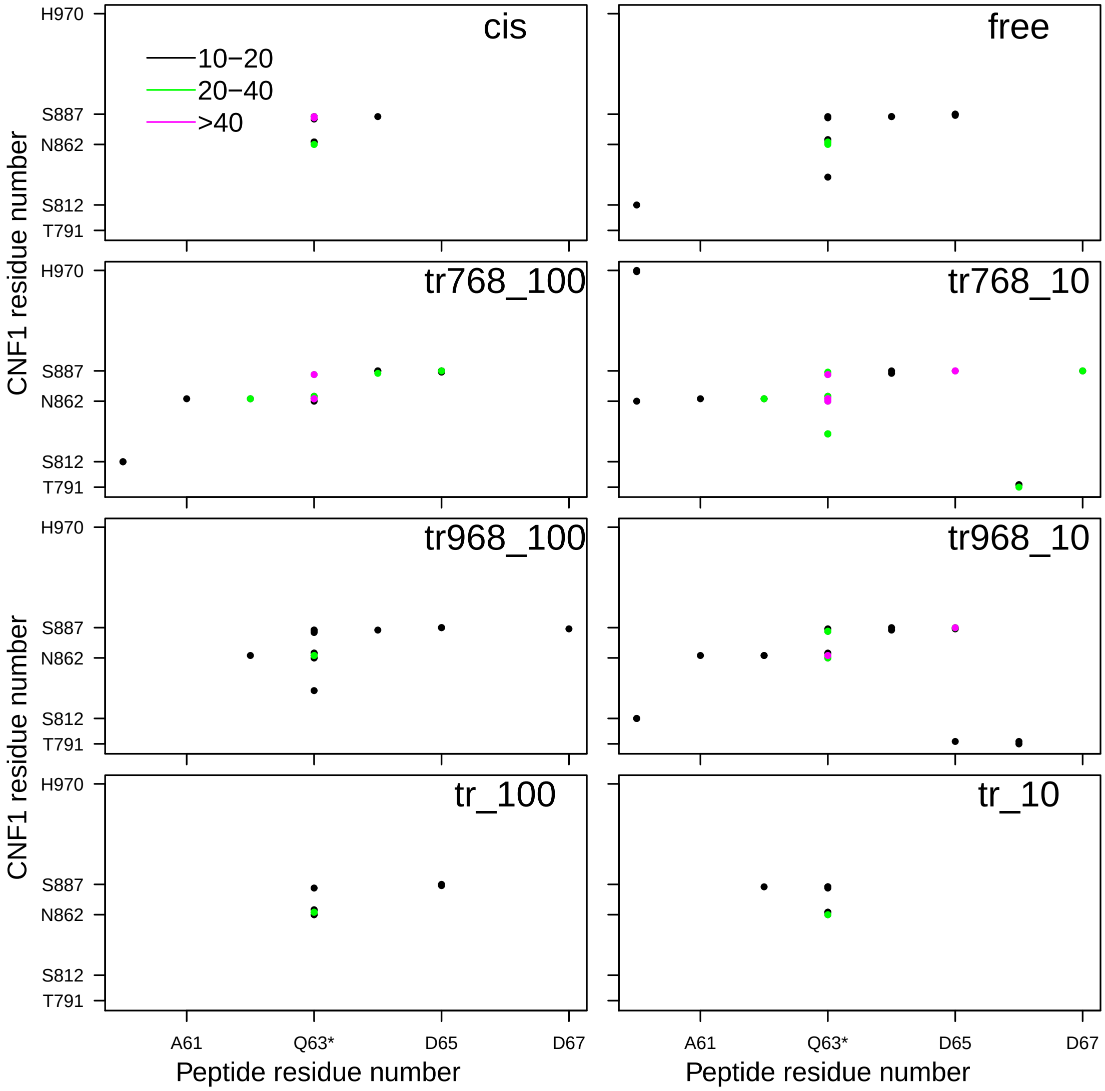

Figure S6: Contact maps of the hydrogen bonds observed between the residues of the SWII domain of RhoA (axis x) and of the CNF1^{CD} domain (axis y) in the various flexpepdock [2] runs. The points on the contact maps are colored depending on the number of hydrogen bonds observed: in the range 10-20 (black), in the range 20-40 (green), larger than 40 (magenta).
